# Expression and functionality of histone H2A variants in cancer

**DOI:** 10.18632/oncotarget.2007

**Published:** 2014-05-26

**Authors:** Fátima Liliana Monteiro, Tiago Baptista, Francisco Amado, Rui Vitorino, Carmen Jerónimo, Luisa A. Helguero

**Affiliations:** ^1^ Mass Specrometry Center, Organic Chemistry and Natural Products Unit (QOPNA), Dep. of Chemistry, Universidade de Aveiro., Aveiro, Portugal; ^2^ Cancer Biology & Epigenetics Group, Research Center Portuguese Oncology Institute – Porto. Porto, Portugal; ^3^ School of Health Sciences, Universidade de Aveiro., Portugal; ^4^ Department of Pathology and Molecular Immunology, Institute of Biomedical Sciences Abel Salazar (ICBAS) – University of Porto, Porto, Portugal

**Keywords:** epigenetics, post-translational modifications, histone H2A, cancer

## Abstract

Regulation of gene expression includes the replacement of canonical histones for non-allelic histone variants, as well as their multiple targeting by postranslational modifications. H2A variants are highly conserved between species suggesting they execute important functions that cannot be accomplished by canonical histones. Altered expression of many H2A variants is associated to cancer. MacroH2A variants are enriched in heterocromatic foci and are necessary for chromatin condensation. MacroH2A1.1 and macroH2A1.2 are two mutually exclusive isoforms. MacroH2A1.1 and macroH2A2 inhibit proliferation and are associated with better cancer prognosis; while macroH2A1.2 is associated to cancer progression. H2AX variant functions as a sensor of DNA damage and defines the cellular response towards DNA repair or apoptosis; therefore, screening approaches and therapeutic options targeting H2AX have been proposed. H2A.Z is enriched in euchromatin, acting as a proto-oncogene with established roles in hormone responsive cancers and overexpressed in endocrine-resistant disease. Other H2A family members have also been found altered in cancer, but their function remains unknown. Substantial progress has been made to understand histone H2A variants, their contribution to normal cellular function and to cancer development and progression. Yet, implementation of high resolution mass spectrometry is needed to further our knowledge on highly homologous H2A variants expression and function.

## INTRODUCTION

Genomic DNA in eukaryotic cells is packaged into chromatin being the nucleosome the smallest subunit. Nucleosomes consist of 145-147 base pairs of DNA wrapped around an octamer of core histone proteins which usually includes two molecules of each of the canonical core histones: H2A, H2B, H3 and H4. These are assembled in one central H3-H4 heterotetramer and two H2A-H2B heterodimers [1, 2] with the linker histone H1 holding the nucleosome together [3]. The highly dynamic changes in nucleosome composition and in their biochemical properties allows regulation of transcription, gene silencing, DNA replication and recombination [4]. Regulation of gene expression at the nucleosome level occurs through combinatorial effects of epigenetic marks including DNA methylation, core histone post-translational modifications (hPTMs) [5] and incorporation of diverse replacement histone variants [6]. While DNA methylation and hPTMs have been the focus of intensive research, much less is known about the mechanisms of core histone replacement and their function. Histones are among the most highly conserved proteins in terms of sequence and structure [7] and replacement of histone variants has been described for all core histone subtypes except histone H4 [4]. Major-type core histones make up the majority of nucleosomes during replication and their expression is tightly coupled to S phase [8, 9]. On the other hand, histone variants are distinct non-allelic forms of core histones, their expression is not restricted to the S-phase, and they are incorporated into nucleosomes through DNA replication-independent mechanisms that often involve specific histone chaperones and ATP-dependent chromatin remodelling factors [10-12]. Substitution of one or more of the core histones with the corresponding non-allelic variants results in differences in nucleosome stability and biochemical properties, thus altering chromatin structure and accessibility of transcription factors and chromatin remodelers to the DNA [13, 14].

Histone variants are highly conserved between different species [15-17], indicating that they have evolved to fulfill important functions that cannot be accomplished by canonical histones [14]. There are several examples of highly divergent replacement variants which have specialized functions and whose deregulation can contribute to cancer development (Table 1). The H2A family of replacement histone variants comprises the largest number of genes identified found associated with cancer, as recently reviewed [18]. H2A variants differ mainly in their N- and C-terminal sequence, whereas the core region is highly conserved [19]. Currently, there are over 19 H2A histone variants identified in human and mouse. They share high degree of homology in their nucleotide and amino acid sequence (Supplementary Tables 1 and 2). This review focuses on H2A histone variants which are altered in cancer; describes their functions, and the methodological difficulties faced in the analysis of many members of the H2A family.

**Table 1:** Histone variants, their known functions and alterations in distinct types of cancer

Histone variant (gene/s)	Proposed function	Altered in cancer
**H1**		
H1.0, H1', H1(0) (*H1F0*)	RNA metabolism [125], control of amphibian and mammalian differentiation [126, 127]	Breast cancer [128], Neuroblastoma [129], Leukemia [130, 131], Melanoma [132], Ovarian cancer [133]
H1.1 (*HIST1H1A*)	Open chromatin [134]	Ovarian cancer [133], Colon cancer [135]
H1.2, H1d (*HIST1H1C*)	Induces apoptosis [136, 137]	Colon cancer [135], Leukemia [138, 139]
H1.3, H1c (*HIST1H1D*)	Promotes chromatin condensation [140] *	Ovarian cancer [133], Breast cancer [112]
H1.4, H1b (*HIST1H1E*)	N/A	Ovarian cancer [133, 141]
H1.5, H1a (*HIST1H1B*)	Binds to families of genes encoding membrane or membrane-related proteins in differentiated cells [142]	Pulmonary neuroendocrine tumor [143]
H1oo, osH1 (*H1FOO*)	Gene expression during oogenesis and early embryogenesis [144]	N/A
H1t (*HIST1H1T*)	Male fertility [145]	Childhood leukemia [139]
H1x (*H1FX*)	N/A	Ovarian cancer [133], neuroendocrine tumors [146]
H1t2 (*H1FNT*)	Spermatogenesis and male fertility [147, 148]	N/A
**H2A**		
mH2A1, H2A.y (*H2AFY*)	X-chromosome inactivation; transcription repression [21, 25, 26, 28, 30]; gametogenesis [149]	Breast cancer [37], lung cancer [39, 40], melanoma [36], colon cancer [38], testicular, bladder, ovarian, endometrial and cervical cancers [40]
mH2A2 (*H2AFY2*)	X-chromosome inactivation [21, 27, 150]	Melanoma [36], lung carcinomas [39]
H2A1, H2A/p, H2A.1 (*HIST1H2AI; HIST1H2AK; HIST1H2AL; HIST1H2AM; HIST1H2AG*)	N/A	Hepatocellular carcinoma [111], colon cancer [135]
H2A1A, H2A/r (HIST1H2AA)	N/A	N/A
H2A1B, H2A.2, H2A/a, H2A/m (*HIST1H2AE; HIST1H2AB*)	N/A	N/A
H2A1C, H2A/I (*HIST1H2AC*)	N/A	Breast cancer [112], lymphocytic leukaemia [113]
H2A1D, H2A.3, H2A/g (*HIST1H2AD*)	N/A	N/A
H2A1H, H2A/s (HIST1H2AH)	N/A	N/A
H2A1J, H2A/e (*HIST1H2AJ*)	N/A	N/A
H2A2A, H2A.2, H2A/o (*HIST2H2AA4; HIST2H2AA3*)	N/A *	Hepatocellular carcinoma [111]
H2A2B (*HIST2H2AB*)	N/A	N/A
H2A2C, H2A-GL101, H2A/q (*HIST2H2AC*)	N/A	N/A
H2A3 (*HIST3H2A*)	N/A	N/A
H2AB1, H2A.Bbd (*H2AFB1*)	Transcription activation [151-153], spermiogenesis [154]	N/A
H2AB2, H2A.Bbd (*H2AFB2; H2AFB3*)	Transcription activation [151-153], Spermiogenesis [154]	N/A
H2A.V, H2A.F/Z (*H2AFV*)	N/A	N/A
H2AJ (*H2AFJ*)	N/A	Melanoma [114], breast cancer [115]
H2A.X (*H2AFX*)	Prevents DNA from double-strand damage [43, 75], apoptosis [63]	Breast cancer [67, 73], lung cancer [68, 72], cervix cancer [71, 72], melanoma [155], leukaemia, colon, ovarian and prostate cancers [70]
H2A.Z, H2AZ, H2A/z (*H2AFZ*)	DNA replication [83], chromosome segregation [86] and maintenance of heterochromatic/euchromatic status [92]	Breast cancer [103, 104, 106], prostate cancer [101, 109], bladder cancer [102], colorectal tumours [99]
**H2B**		
H2B1A (*HIST1H2BA*)	Testis-specific, Chromatin integrity [156]	N/A
H2B1B (*HIST1H2BB*)	N/A	N/A
H2B1C, H2B.1A, H2B.a, H2B.g, H2B.h, H2B.k, H2B.i (*HIST1H2BG; HIST1H2BF; HIST1H2BE; HIST1H2BI; HIST1H2BC*)	N/A	Breast cancer [112]
H2B1D, H2B.1B, H2B.b (*HIST1H2BD*)	N/A	N/A
H2B1H, H2B.j (*HIST1H2BH*)	N/A	N/A
H2B1J, H2B.1, H2B.r (*HIST1H2BJ*)	Chromatin remodelling in schizophrenia [157]	N/A
H2B1K, HIRA-interacting protein 1 (*HIST1H2BK*)	N/A	N/A
H2B1L, H2B.c (*HIST1H2BL*)	N/A	Gastric cancer [158]
H2B1M, H2B.e (*HIST1H2BM*)	N/A	Breast cancer [159]
H2B1N, H2B.d (*HIST1H2BN*)	N/A	N/A
H2B1O, H2B.2, H2B.n (*HIST1H2BO*)	N/A	Breast cancer [112], acute myeloid leukemia [160]
H2B2E, H2B-GL105, H2B.q (*HIST2H2BE*)	Inhibits cell proliferation [161], inactive odour-sensing neurons [162]	Gastric cancer [161]
H2B2F (*HIST2H2BF*)	N/A	Prostate cancer [163]
H2B3B, H2B type 12 (*HIST3H2BB*)	N/A	N/A
H2BFM, H2B.s (*H2BFM*)	N/A	N/A
H2BFS, H2B.s (*H2BFS*)	N/A	N/A
H2BWT (H2BFWT)	Telomeric preservation during mitosis (85)	N/A
H3		
H3.1 (*HIST1H3A; HIST1H3D; HIST1H3C; HIST1H3E; HIST1H3I; HIST1H3G; HIST1H3J; HIST1H3H; HIST1H3B; HIST1H3F*)	DNA replication and repair [164], cell differentiation [165]	Colon cancer [135]
H3.2, H3/m, H3/o (*HIST2H3C; HIST2H3A; HIST2H3D*)	N/A	Colon cancer [135]
H3.3 (*H3F3A; H3F3B*)	Transcription activation (165, 166, 167)	Acute myeloid leukemia [160], breast cancer [166]
H3.1t, H3/g, H3t, H3/t, (*HIST3H3*)	Chromatin reorganization during meiosis and/or spermatogenesis [167]	N/A
H3.3C, H3.5 (*H3F3C*)	Active chromatin [168]	N/A
CENP-A (*CENPA*)	Kinetochore assembly [169-171]; mitosis [172]	Human testicular germ cell tumours [173], hepatocellular carcinoma [174], colorectal cancer [175], breast cancer [176]

### macroH2A histone variants

**Figure 1:**
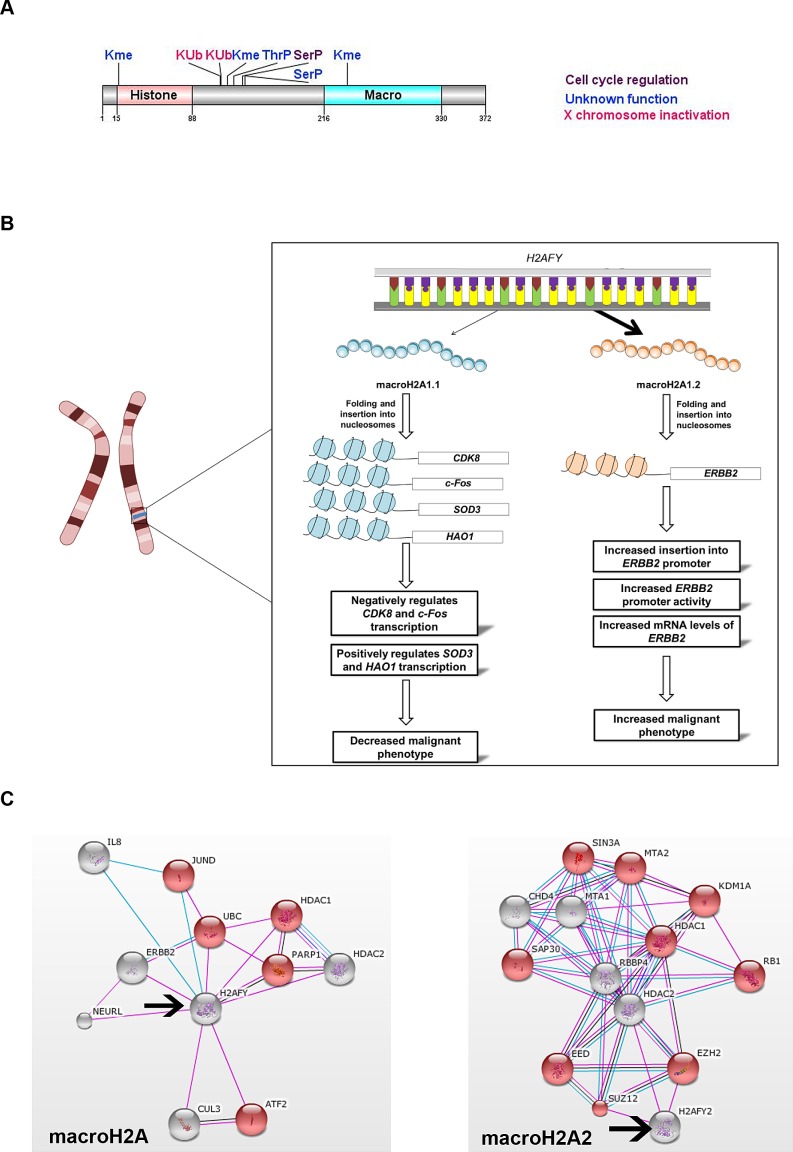
Role of *H2AFY* and *H2AFY2* in cancer progression A. Structural domains and postranslational modifications identified on macroH2A.1 protein [22-25]. B. Alternative splicing of *H2AFY* gives rise to macroH2A1.1 and macroH2A1.2 isoforms. Their incorporation into nucleosomes of specific genes exerts different effects. MacroH2A1.1 negatively impacts cancer progression through inhibition of expression of genes that stimulate cell proliferation (*CDK8* and *c-Fos*) and involved in redox metabolism (*SOD3* and *HAO1*).On the othe hand, macroH2A1.2 substitution in *ERBB2* oncogene promoter stimulates its expression. C. Protein interaction network obtained from String DB (string-db.org) illustrates one of the possible biological processes regulated, as inferred from analysis of public databases. Both macroH2A variants (arrows) can differentially interact with histone modifying proteins and transcription factors which results in regulation of a variety of biological processes. One differential regulation is highlighted in red, where macroH2A1 interactors influence gene expression (p<0.016), while macroH2A2 inhibit transcription (p<1.05 × 10^−5^). Grids were obtained using a confidence score of 0.6. Colour key: pink = experimental evidence; blue = evidence from databases; black = co-expression.

MacroH2A is an extremely divergent H2A variant with a tripartite structure consisting of an amino-terminal histone-like region that is 64% identical in the amino acid

sequence to full length histone H2A, a large carboxyl-terminal globular domain and the macro domain which is a lysine (K) rich H1-like linker region that includes a random coil with no similarity to other histones [15, 20]. Two macroH2A genes are present in vertebrates, *H2AFY* and *H2AFY2*, which encode macroH2A1 and macroH2A2 proteins, respectively [21]. Several PTMs have been identified on histone macroH2A1, mostly in the N-terminal domain (Fig. 1A) [22-25].

Constanzi *et al.* originally found macroH2A1 as enriched in inactive X chromosome (*Xi*) in female mouse, dog and human [26]. A few years later, macroH2A2 was also identified in *Xi* chromosome [21, 27]. MacroH2A1 occupies 25% of the human genome and is incorporated into nucleosomes found in heterochromatin. Its ubiquitination by the CULLIN3/speckle-Type POZ Protein (SPOP) E3 ligase complex results in stable X chromosome inactivation in mammalian females [25]. Several independent observations support the idea that the enrichment of macroH2A in the nucleosome correlates with heterochromatin and gene silencing. Namely, macroH2A interferes with transcription factor binding and nucleosome remodelling by SWI/SNF complexes [28]; enrichment of macroH2A1 is associated to the facultative H3K27me3 heterochromatin mark and to the depletion of active transcription marks such as RNA polymerase II, H3K4me1, and histone H3 acetylation; macroH2A1 is mainly localized near transcription start sites (TSSs) and CTCF-binding sites [29] and is enriched in transcriptionally silent senescence-associated heterochromatic foci [30]. MacroH2A proteins constitute a repressive mark that contributes to the fine-tuning of temporal activation of *HOXA* cluster genes during neuronal differentiation and its loss in zebrafish embryos leads to severe developmental defects [31]. Therefore, macroH2A variants constitute an important epigenetic mark involved in the concerted regulation of gene expression programs during cellular differentiation and vertebrate development [31]. On the other hand, macroH2A can be phosphorylated by Cdk complexes, containing cyclin E and cyclin B. Consequently, macroH2A-phSer137 is excluded from heterochromatin in Xi chromosome and is enriched in heterochromatin during mitosis, suggesting it may play a role in cell cycle regulation [22].

Contrary, to its enrichment in heterochromatin, macroH2A positively regulates a subset of specific genes associated with lipid metabolism during liver transition from newborn to young-adult state [32]. In line with this work, macroH2A1 is likely found in promoter-proximal regions in IMR90 human primary lung fibroblasts and MCF-7 breast cancer cells and can also increase signal-regulated transcription, specifically for genes activated by serum starvation [29].

The *H2AFY* gene contains two mutually exclusive exons which can be alternatively spliced to originate two isoforms, macroH2A1.1 and macroH2A1.2 (Fig. 1B) [27, 33], differing only in a part of the non-histone region [34]. MacroH2A1.1 splice variant is mostly restricted to differentiated cells [33, 34] and can bind to nicotinamide adenine dinucleotide (NAD)^+^-derived ligands; while macroH2A1.2 cannot interact with these small molecules [35], it is generally expressed in embryonic stem cells and the early embryo [27, 33, 34].

### Histone macroH2A in cancer

MacroH2A variant expression is majorly lost in melanoma progression, mainly through promoter methylation, as shown for macroH2A2 in metastatic disease [36]. In addition, macroH2A1 suppresses melanoma progression through downregulation of cyclin-dependent kinase 8 (CDK8) gene expression, which promotes cellular proliferation through enhancement of malignant transformation by β-catenin [36]. While in normal adult cells macroH2A1 isoforms are expressed with similar levels, macroH2A1.1 decreases in a variety of human cancers including breast [37], colorectal [38], lung [39, 40], testis, bladder, ovarian, endometrial and cervical cancers [40]; and the ratio macroH2A1.1 / macroH2A1.2 has a profound effect on patient prognosis and survival. In cancers with poor prognosis, alternative splicing of the *H2AFY* gene strongly favours macroH2A1.2 expression, due to decreased activity of the splicing factor QKI [39, 40]. MacroH2A1.1 is highly expressed in tumours with better prognosis and low mitotic index [39]; while its low levels correlate to tumours undergoing rapid cell division marked by high expression of the proliferation marker Ki-67 [39]. Moreover, macroH2A1.1 downregulation favours metastasis and correlates with decreased patient survival and tumour recurrence, as shown in lung cancer [39]. Furthermore, macroH2A1.1 is upregulated in senescent cells and triggers oncogene-induced senescence [30].

Poly (ADP-Ribose) Polymerase 1 (PARP-1) has been implicated in several processes that promote cellular proliferation of lung and cervical cancer cells. Their growth suppression is at least in part, mediated by macroH2A1.1 interaction with PARP-1 and its subsequent downregulation [40]. MacroH2A1.1 not only acts as a transcriptional repressor as in the case of the oncogene *c-Fos* [41], but it also promotes the expression of proteins involved in redox metabolism, such as Superoxide Dismutase 3, Extracellular (*SOD3)*, Hydroxyacid Oxidase (Glycolate Oxidase) 1 (*HAO1*), Rieske (Fe-S) Domain Containing (*RFESD*) and Glucose-Fructose Oxidoreductase Domain Containing 1 (*GFOD1)* [42] (Fig. 1B). Depletion of macroH2A1.2 in metastatic 4T1 cells, which under normal conditions display a higher content of macroH2A1.2 in detriment of macroH21.1, induces *SOD3* expression. In a similar way, depletion of macroH2A1.1 in non-metastatic cells 67NR, which have a high macroH2A1.1/macroH2A1.2 ratio, *SOD3* was also induced, suggesting that macroH2A1.1 is able to promote *SOD3* expression, while macroH2A1.2 inhibits it [42]. Also, macroH2A1.2 is by far the predominant form in MCF-7 breast cancer cell line [29] and can interact with HER-2 in the nucleus to enhance the over-expression of oncogene *ERBB2* [37] (Fig. 1B). Therefore, while it is generally accepted that histones macroH2A1.1 and macroH2A2 act as tumour suppressors, macroH2A1.2 variant seems to be an oncogene associated with disease progression and negative patient outcome.

The varying array of biological processes regulated by macroH2A histones can be inferred from their network of interacting proteins including positive and negative regulators of transcription (Fig. 1C). Interestingly, one of the major advantages of cancer cells compared to non-malignant cells is their ability to adapt their metabolism to the nutrient availability. Thus, identification and understanding the macroH2A protein complexes that regulate genes in redox metabolism such as *SOD3* as well as lipid metabolism genes deserves further study.

### H2AX histone variant

H2AX levels are cell line or tissue specific and represent about 2–25% of total H2A [43]. In contrast to other genes encoding H2A histone variants, *H2AFX* contains landscapes of both replication dependent and replication-independent histone species [44]. It has been proposed that this dual mechanism of translational regulation ensures the presence of sufficient H2AX molecules in the replicating genome for efficient and continued presence of H2AX at G1 and G0 phase of the cell cycle [45].

**Figure 2:**
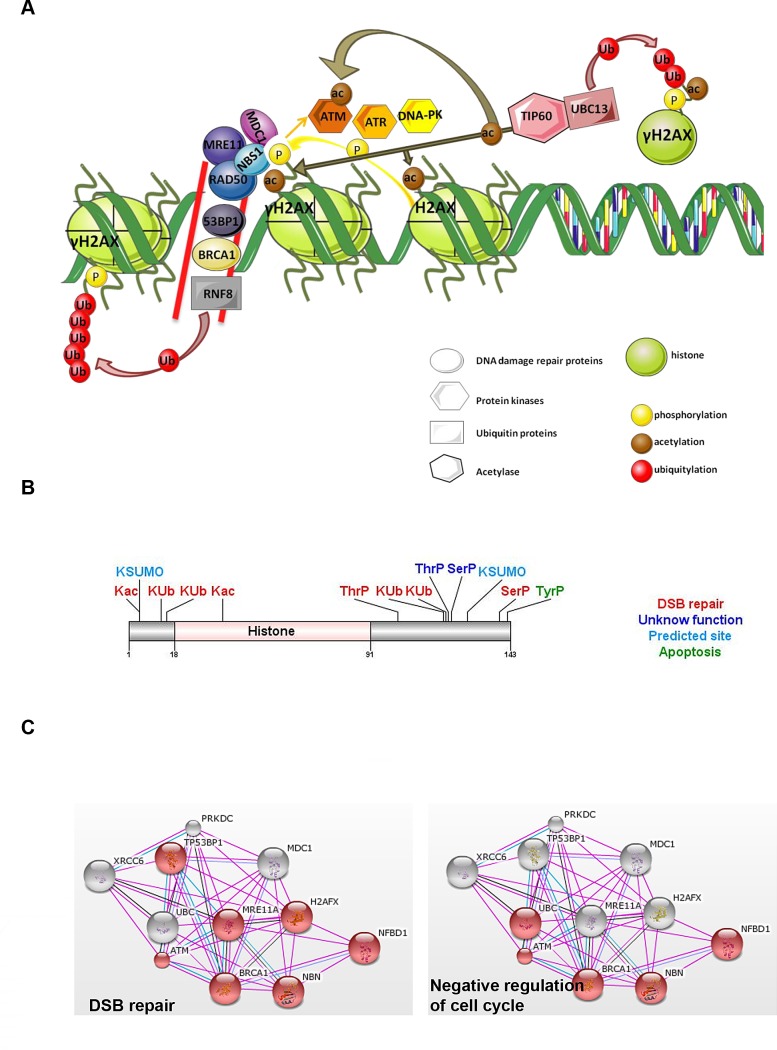
H2AX histone variant A. Main mechanisms involved in DNA damage repair system (DDR). Upon DNA double strand breaks (DSB; signalled by two red lines), H2AX is recruited and phosphorylated in serine 139 (γH2AX) by PIKKs, ATM and ATR protein kinases. NBS1 and MDC1 binding to γH2AX amplify H2AX phosphorylation through stimulation of ATM and also induce recruitment of DDR repair proteins to sites of DSBs. TIP60 and UBC13 activate H2AX through acetylation and independently of phosphorylation. Acetylated H2AX is then released from chromatin and ubiquitylated (Ub) by UBC13 and RNF8, leading to formation of ubiquitin chains and recruitment of various DDR proteins containing ubiquitin-binding domains. B. Summary of H2AX protein domain and the multiple regulatory PTMs identified. [47, 48, 55, 58, 59, 64, 65, 123, 124]. All PTMs related with DSB repair have an indirect role associated with cancer. C. Protein interaction network obtained from String DB (string-db.org) illustrates one of the possible biological processes regulated, as inferred from analysis of public databases. In this case, the protein interaction network shows how different partners regulate H2AX effects on biological processes highlighted in red: double strand break repair (DBS; p<1.9 × 10^−12^) or cell cycle (p<1.04 × 10^−4^). Grids obtained from String DB (string-db.org) using a confidence score of 0.6. Colour key: pink = experimental evidence; blue = evidence from databases; black = co-expression.

The main function of H2AX is associated with the DNA damage repair (DDR) system which is induced by DNA double strand breaks (DSBs) (Fig. 2A), being H2AX function regulated by multiple PTMs (Fig. 2B). One of the first responses to DSBs in eukaryotic cells is the phosphorylation of serine 139 in the C-terminal tail of histone H2AX. This yields a specific modified form known as γH2AX, and promotes recruitment of DNA repair proteins to sites of DSBs [43, 46], leading to a linear increase in the number of γH2AX molecules with the severity of the DNA damage. Therefore, γH2AX has been used as a sensitive marker for the presence of DSBs in cells and tissues [43]. H2AX can also be acetylated in K36 by the CBP/p300 acetyltransferases [47] and in threonine (Thr) 101 [48]. These two PTMs are required for cells to survive exposure to ionizing radiation (IR) independently of H2AX phosphorylation [48].

H2AX phosphorylation is catalysed by three phosphatidylinositol-3 kinase-like kinases (PIKKs): ataxia telangiectasia mutated (ATM), ataxia telangiectasia and Rad3-related (ATR) and DNA-dependent protein kinase (DNA-PK) [49, 50]. A signal amplification loop involving H2AX, Nijmegen breakage syndrome 1 (nibrin- NBS1) and mediator of DNA damage checkpoint protein 1 (MDC1) stimulates ATM and increases H2AX phosphorylation [51]. NBS1 and MDC1 bind directly to γH2AX through MDC1 BRCT domain, which allows the accumulation of DDR proteins including the MRN (MRE11–RAD50–NBS1) complex, Ring Finger Protein 8, E3 Ubiquitin Protein Ligase (RNF8), Breast Cancer 1, Early Onset (BRCA1) and p53-binding protein 1 (53BP1) [52-54]. In addition, DSBs facilitate TIP60 and Ubiquitin-Conjugating Enzyme E2N (UBC13) association and further ATM acetylation and activation as well as H2AX acetylation in K5-independently of its phosphorylation [55]. Consequently, H2AX is released from chromatin and it is ubiquitylated on K119 by UBC13 [55]. Ring Finger Protein 168, E3 Ubiquitin Protein Ligase (RNF168) and RNF8 also ubiquitylate H2AX on K13, K15, K118 and K119, which initiate the formation of ubiquitin chains and recruitment of various DDR proteins containing ubiquitin-binding domain [55-60]. The H2AX ubiquitination by RNF168 and RING1B/BMI is mediated by the nucleosome acid patch which is also required for RNF168- and RING1B/BMI- dependent DDR proteins recruitment to repair DNA damage [61]. Although it is generally accepted that DNA DSBs induce the formation of γH2AX foci, DNA single-stranded regions induced by ultraviolet C irradiation can also induce formation of γH2AX [50]. Furthermore, in apoptosis provoked by UV irradiation, γH2AX may be further phosphorylated by c-Jun NH2-terminal kinase (JNK) [62] and function as a response to endonuclease-mediated DNA fragmentation downstream from caspase-3/caspase-activated DNase (CAD) pathway activation [63]. More recent studies reveal that the decision to undergo DDR or apoptosis is determined by phosphorylation of γH2AX in tyrosine 142 (Tyr142P)[64, 65]. As it inhibits the binding of repair factors to γH2AX and promotes the recruitment of pro-apoptotic factors such as JNK1 [64]. On the other hand, loss of H2AX Tyr142P, alters the kinetics of γH2AX in response to DNA damage [65].

### Histone H2AX in cancer

The association of DNA damage, apoptosis and genome stability with premalignant stages and progression of a tumour is highly recognized. *H2AFX* is located in a chromosome region that frequently exhibits mutations or deletions in a large number of human cancers (11q23), especially in haematopoietic malignancies [66]. Furthermore, a reduction in *H2AFX* gene copy number was verified in MCF7 breast cancer cell line [67] and higher methylation status of *H2AFX* promoter leads to a reduction of H2AX expression in lung squamous cancer [68]. Further, upregulation of H2AX by the clinically approved protein kinase inhibitor Imatinib mesylate triggers apoptosis in gastrointestinal stromal tumour cell lines [69].

Based on H2AX function in DNA repair and in maintaining DNA stability, the use of H2AX / γH2AX as marker for early cancer detection, prognosis and therapeutics has been proposed [70]. Elevated endogenous levels of γH2AX have been found in various human cancer cell lines such as cervical [71], ovarian [72], breast [73], leukaemia and melanoma, colon, renal, and prostate cancer cell lines [70]. Many therapeutic agents act by introducing sufficient DSBs into cancer cells to activate the apoptotic pathway [74]. Thus, H2AX could also be targeted to promote cancer cell death. For example, latrunculin B, an agent that inhibits actin dynamics, induces γH2AX formation, leading to G2 arrest and consequently resulting in MCF-7 breast cancer cell apoptosis [75]. Therefore, quantification of γH2AX enriched foci to detect DSBs formation, may be a sensitive method to monitor either cancer progression or response to treatment [70].

### H2A.Z histone variant

The variant H2A.Z is highly conserved from yeast to human, with 90% of its primary sequence preserved among different species, showing only 60% homology with canonical histone H2A [76]. H2A.Z has been one of the most studied H2A variants in recent years. Different studies reported diverse and controversial conclusions regarding alteration in the nucleosome stability by insertion of this variant. Some authors claim that H2A.Z nucleosomes are more stable [77], while others have observed that H2A.Z incorporation destabilizes nucleosome core particles [13, 78]. In yeast, the mechanism by which histone H2A is replaced by H2A.Z in the nucleosome is mediated through the action of a multisubunit protein complex, SWR1-Complex, which contains a Swi2/Snf2 paralog [79]. In higher eukaryotes, two SWR1-related multiprotein complexes (SRCAP and p400/TIP60) were described. While the SRCAP chromatin remodeling complex is involved in global H2A.Z deposition activity [80], the p400/TIP60 complex is known to mediate a more localized deposition of H2A.Z [81]. Recently, it was described that the acidic nuclear phosphoprotein 32 kilodalton E (ANP32E), a member of the p400/TIP60 complex, is responsible for the removal and deposition of H2A.Z in the nucleosome [82].

**Figure 3:**
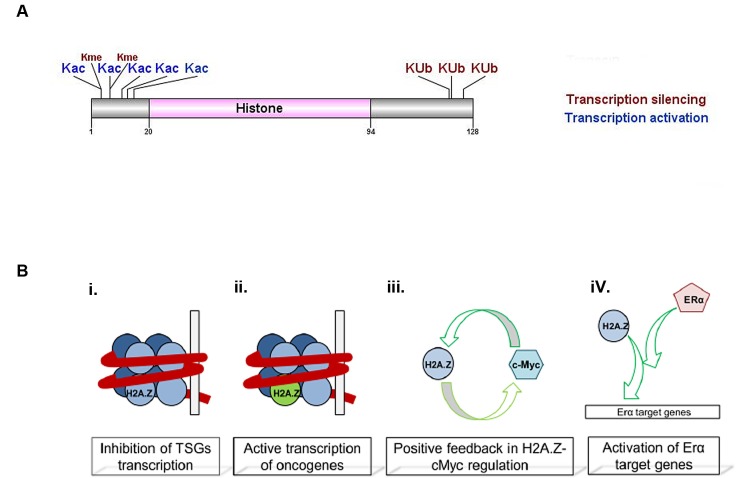
H2A.Z histone variant structure and function A. H2A.Z protein domain and reported PTMs [93, 94, 97, 98, 109]. Those hPTMs with a function in cancer are in larger font. B. H2A.Z in cell proliferation. Incorporation of non-acetylated H2A.Z into nucleosomes inhibits tumor suppressor genes (TSG) transcription (i) and is found in actively transcribed oncogenes in its acetylation form (ii). One example is H2A.Z enrichment in c-Myc promoter, which stimulates its expression, leading to higher c-Myc protein levels which in turn activates H2A.Z transcription (iii). H2A.Z is found enriched in ERα target genes and is necessary for ERα transactivation of proliferation genes in breast cancer (iv).

H2A.Z is found in approximately 10% of mammalian nucleosomes and participates in different biological processes such as cell cycle and DNA replication [83], DNA repair [84], spermatogenesis [85], chromosome segregation [86], centromere structure [87], transcription regulation [88-91], and maintenance of heterochromatic/euchromatic status [92, 93]. However, its role in transcriptional regulation is complex since it has been reported to function both as a transcriptional repressor and activator. The apparently contradictory roles of H2A.Z *in vivo* might be explained by different combinations of H2A.Z with other epigenetic regulators, PTMs on H2A.Z (Fig. 3A) and interaction with chromatin binding proteins (Fig. 2B) [94]. Acetylated H2A.Z is enriched at the 5' regions of active genes in yeast and vertebrates [95, 96], whereas ubiquitylated H2A.Z associates with facultative heterochromatin [97], and monomethylation of H2A.Z at K7 by the lysine methyltransferase SETD6 has been suggested as a marker of cellular differentiation [98].

### Histone H2A.Z in cancer

A possible role for H2A.Z in cancer was first reported using genome wide gene expression profiling where overexpression of H2A.Z was observed in sporadic colorectal tumors [99]. Undifferentiated cancers show *H2AFZ* overexpression compared to well differentiated cancers [100] and overexpression of this histone variant was also reported in genitourinary cancers, such as prostate [101] and bladder cancer [102]. Breast cancer is where H2A.Z role has been best characterized, with its overexpression correlating with lymph node metastasis in primary breast cancer [103], and overexpression also observed in late stages of the disease [104]. The correlation between H2A.Z levels and short patient overall survival suggests that this histone variant might be a biomarker of tumor progression.

H2A.Z gene is under the positive control of c-Myc (Fig. 3B) and therefore might be an important indirect target for breast cancer therapy [105]. H2A.Z positively regulates estrogen receptor (ER)α-dependent transcription and estrogen simulation of cell proliferation [106]. Yet, in MCF7 cells, H2A.Z overexpression also promotes cellular proliferation under low estrogen levels and upon treatment with the ER antagonist tamoxifen, which suggests that proliferation induced by H2A.Z overrides the inhibitory effects of tamoxifen on gene transactivation by ERα and may play a role in endocrine resistance [107].

H2A.Z is also associated to androgen receptor (AR) gene transactivation and progression of prostate carcinoma (PCa). H2A.Z ubiquitylation in K120, K121 and K125 has been described as present in PCa and associated with transcriptional silencing (Fig. 3A). Deubiquitylation of H2A.Z by ubiquitin-specific protease (USP10) leads to transcriptional activation of the AR-regulated *PSA* and *KLK3* genes [97]. A significant increase of H2A.Z was found in castration-resistant LNCaP xenograft model [108]. Furthermore, since PCa patients submitted to androgen-deprivation therapy tend to express more H2A.Z over time, it has been suggested that the elevated expression of H2A.Z might be indicative of primary PCa progression to androgen-independence [108]. A recent study in PCa cell lines evaluated how H2A.Z and its acetylation in K4, K7 and K11 (acH2A.Z) positively regulates transcription of oncogenes and showed that acH2A.Z mutually excluded DNA methylation and the deposition of the H3K27me3 mark within the promoter region [109]. Consequently, acH2A.Z tended to accumulate within the TSS of active genes and was tightly associated with active gene transcription [109]. In line with this, H2A.Z deacetylation is most prevalent in nucleosomes next to the TSS and correlates with lower gene transcription activity including that of tumor suppressor genes [109]. More recently, Baptista, *et al.* using PCa cell lines showed that H2A.Z regulates its own expression by increasing its accumulation nearby the TSS of the *H2AFZ* gene, while its regulation is impaired by decreased expression and protein levels of the histone deacetylase NAD-dependent protein deacetylase sirtuin-1 (SIRT1), which is necessary to maintain H2A.Z levels [101]. Furthermore, effective restoring of SIRT1 function by epigenetic modifying drugs in conjunction with enzymatic modulators lead to proteasomal degradation of H2A.Z and of its target/regulator c-Myc. Therefore, SIRT1 activation, emerged as a promising tool for targeted therapy of endocrine-resistant PCa patients through reduction of H2A.Z [101].

A dependence of H2A.Z for cancer cell proliferation, viability and progression into cell cycle was showed in the osteosarcoma U2OS cell line with H2A.Z depletion; However, the same was not observed in relation to DNA repair [110]. These results seems to be contradictory, since Xu et al described that H2A.Z exchange at DSBs shifts the chromatin to an open conformation required for loading some of the DDR proteins [84]. The authors suggested that the p53 status and the cell line may be key to explain these contradictory results [110].

### Other H2A variants in cancer

Additionally, there are some H2A variants altered in cancer but with no associated function identified. For example, during sequential development of hepatocellular carcinoma, the major histone H2A variant H2A.1 *(HIST2H2AA1)* is overexpressed, and H2A.2 (*HIST2H2AA3*) is decreased at the protein and mRNA levels [111]. The histone variant H2A1C was described as overexpressed in MCF-7 cell line along with the silencing of the oncogenic protein phosphatase magnesium-dependent 1 delta (PPM1D) [112]. Moreover, in acute myeloid leukemia, reduced expression of the *HIST1H2AC* locus leads to increased rates of cell proliferation and tumorigenicity [113], also supporting a loss of function for H2A1C during cancer progression. Lastly, H2AFJ has been subject of controversial findings, with downregulation reported in melanocytic tumor lesions [114] and overexpression in breast cancers with 12p13 regional copy number gain compared with a panel of normal mammary epithelial cells [115].

### Future challenges in the study of H2A family members

Methods using antibodies or DNA probes are the primary tools used for molecular and biochemical investigation. Since many members of the H2A family share high sequence homology (Supplementary Tables 1 and 2), the lack of reagents with high-specificity for individual variants has hindered studies on expression and function of several H2A variants. Some variants share up to 98% homology and therefore, even approaches designed to identify non-homologous regions are restricted. The high homology in the base pair sequence and existence of duplicated genes poses a methodological draw-back for the design of probes for RNA quantification, alignment of RNA-seq data and gene expression silencing using siRNAs. In addition, the high similarity of epitopes in H2A variants and the variety of PTMs on these molecules complicate the generation of specific antibodies adding to the difficulty of finding high quality antibodies for H2A variant quantification and chromatin immunoprecipitation (ChIP / ChIP-Seq) analysis. Consequently, and not surprisingly, research has concentrated on those variants which are less conserved and for which it has been easier to obtain specific detection reagents.

Mass spectrometry (MS) has become widely used to analyze histone variants. MS has an advantage over the limitations posed by immunological reagents and has emerged as a promising complementary analytical strategy not only to identify known and novel PTMs on proteins, but also for their relative quantification [116-119]. Yet, MS also has its limitations. For instance, some histone H2A family members differ in sequence by as little as one amino acid residue which can result in false positive identifications by attribution of multiples of 14 Da mass shifts due to the amino acid differences between the variants which is also traditionally assigned as methylation [120]. The recent advent of high-resolution mass spectrometry (possible with modern mass spectrometers, with a resolution that allows accurate determination of the mass corresponding to a molecular ion), in combination with different strategies for peptide fragment dissociation, electron capture dissociation (ECD) and electron transfer dissociation (ETD) has increased the relevance of MS-based PTM characterization in unveiling the histone code [121]. Indeed, the high mass accuracy afforded by high-resolution MS data greatly increases the confidence in assigning a protein modification. For example, the difference in mass (Δm) of 14 Da, attributed to Glu->Asp and also to an aminoacid methylation, can be discerned using high-resolution MS since Glu->Asp presents a Δm 14.015650 Da) and methylation presents a Δm 14.016650 Da). Furthermore, histone variant analysis is highly dependent on liquid chromatography separation whose use is critical in the case of modified histone peptides from a complex sample mixture of a wide concentration range -including large peptides with identical amino acid sequences modified in slightly different ways- and which result in many isobaric structural isomers [122]. Nevertheless, despite these drawbacks, the high throughput tools available in MS labs allow a sensitive and reproducible histone profiling that will be of great value for exploring variants and their PTMs and which can readily be applied to clinical or pharmaceutical studies.

In summary, the immunological limitations for studying the expression and function of H2A variants make it a challenging field of research. There has been considerable progress made, yet overcoming these difficulties will require improving combinatorial mass spectral methods to bypass the necessity for immunological reagents. In addition, site-directed mutagenesis is also one option for functional analysis to uncover the specific cellular functions of each H2A variant and their respective PTMs.

## CONCLUSION

Substitution of canonical H2A by its non-allelic variants modifies nucleosome biophysical properties, chromatin structure and function. Epigenetic alterations preceding cancer disease or evolving alongside progression appear related to H2A variant replacement. Further, PTMs, at the inter-phase of histone function and histone differential protein interactions with chromatin remodelers and transcription factors contribute to expression of genes important for DNA repair, redox metabolism, proliferation, survival and metastasis. Much progress has been made in understanding the functions of several H2A variants and to describe their alterations in cancer. These results lead to propose non-canonical H2A variants as markers of disease progression and response to cancer therapy. Notwithstanding the difficult task of finding highly specific antibodies, future work should be done to validate these data in a broader number of cancer cases, as well as developing highly sensitive MS-based methodologies to discriminate H2A variants with high sequence homology and for which specific detection reagents are lacking.

## SUPPLEMENTARY TABLES



## List of Abbreviations

Ac: acetylated
ANP32E: acidic nuclear phosphoprotein 32 kilodalton E
AR: androgen receptor
ATM: ataxia telangiectasia mutated
ATR: ataxia telangiectasia and Rad3-related
BRCA1: Breast Cancer 1, Early Onset
CAD: caspase-activated DNase
CDK8: cyclin-dependent kinase 8
c-Fos: FBJ Murine Osteosarcoma Viral Oncogene Homolog
ChIP: chromatin immunoprecipitation
DDR: DNA damage repair
DNA-PK: DNA-dependent protein kinase
DSBs: DNA double strand breaks
*ERBB2*: V-Erb-B2 Avian Erythroblastic Leukemia Viral Oncogene Homolog 2
Eya: eyes absent homolog
*GFOD1*: Glucose-Fructose Oxidoreductase Domain Containing 1
*HAO1*: Hydroxyacid Oxidase (Glycolate Oxidase) 1
hPTMs: histone post-translational modifications
IR: ionizing radiation
JNK: c-Jun NH2-terminal kinase
K: lysine
MDC1: mediator of DNA damage checkpoint protein 1
MS: Mass spectrometry
NAD: Nicotinamide adenine dinucleotide
NBS1: Nijmegen breakage syndrome 1 (nibrin)
P: phosphorylation
PARP-1: Poly (ADP-Ribose) Polymerase 1
PCa: prostate carcinoma
PIKKs: phosphatidylinositol-3 kinase-like kinases
PPM1D: oncogenic protein phosphatase magnesium-dependent 1 delta
*RFESD*: Rieske (Fe-S) Domain Containing
RNF8: Ring Finger Protein 8, E3 Ubiquitin Protein Ligase
Ser: serine
*SOD3*: Superoxide Dismutase 3, Extracellular
SPOP: speckle-Type POZ Protein
Thr: threonine
TSSs: transcription start sites
Tyr: tirosine
Ub: ubiquitylation
UBC13: Ubiquitin-Conjugating Enzyme E2N
USP10: ubiquitin-specific protease
*Xi*: inactive X chromosome
53BP1: p53-binding protein 1
