# Supplementary material for: Expression and functionality of histone H2A variants in cancer
Source: Oncotarget. 2014 May 26;5(11):3428–43. doi: 10.18632/oncotarget.2007 (PMC4116493; doi:10.18632/oncotarget.2007)
Supplement: Supplementary file 1 [file oncotarget-05-3428-s001.pdf]

Expression and functionality of histone H2A variants in cancer

Supplementary Table 1:Nucleotide and amino acid sequence homology of human H2A family members

HUMAN				
	Nucleotide		Protein	
Histones	Name of coding gene(s)	Homology*	Name (s)	Homology**
H2A histone family, member Y	H2AFY, MACROH2A1		mH2A1, macroH2A1, H2A.y, H2a/y, macro-H2A.1, macroH2A1.1, macroH2A1.2	68% with mH2A2<br>68% with H2AX<br>68% with H2A2B<br>67% with H2A1A<br>67% with H2A2C<br>67% with H2A2A<br>66% with H2A.J<br>66% with H2A3<br>66% with H2A1C
	H2AFY2, MACROH2A2		mH2A2, macroH2A2, macro-H2A.2	68%% with mH2A1<br>67% with H2A2B<br>66% with H2A1A<br>66% with H2A2C<br>66% with H2A2A
H2A histone family, member J	H2AFJ	85% with HIST1H2AK<br>85% with H2AFX<br>85% with HIST1H2AI	H2A.J, H2a/j	98% with H2A2A<br>98% with H2A.1<br>98% with H2A1H<br>98% with H2A1C<br>97% with H2A2C<br>97% with H2A1J<br>97% with H2A2B<br>97% with H2A1D<br>97% with H2A3<br>96% with H2A type 1-B/E<br>95% with H2AX<br>91% with H2A1A<br>67% with H2A.V<br>66% with mH2A1
H2A histone family, member V	H2AFV, H2AV		H2A.V, H2A.F/Z	98% with H2A.Z<br>66% with H2A2C<br>66% with H2A2A<br>66% with H2A3<br>66% with H2A1D

H2A histone family, member X	H2AFX, H2AX	88% with HIST3H2A<br>87% with HIST1H2AH<br>87% with HIST1H2AD<br>86% with HIST1H2AI<br>86% with HIST1H2AL<br>85% with HIST1H2AK<br>85% with HIST2H2AC<br>85% with HIST2H2AA3<br>85% with HIST2H2AA4<br>85% with HIST1H2AG	H2AX, H2a/x	96% with H2A2B<br>96% with H2A1C<br>95% with H2A2C<br>95% with H2A.J<br>95% with H2A2A<br>95% with H2A1J<br>95% with H2A.1<br>95% with H2A1H<br>95% with H2A3<br>94% with H2A1D<br>94% with H2A1B<br>93% with H2A1A	

				68% with mH2A1<br>66% with mH2A2
H2A histone family, member Z	H2AFZ, H2AZ		H2A.Z, H2A/z	98% with H2A.V
H2A histone family, member B	H2AFB1	99% with H2AFB2<br>99% with H2AFB3	H2A-Bbd type 1, H2A<br>B b d d fi i t<br>H2A.Bbd	99% with H2A-Bbd type 2 /
	H2AFB2; H2AFB3	99% with H2AFB1	H2A-Bbd type 2/3, H2A<br>H2A.Bbd	99% with H2A-Bbd type 1
	HIST1H2AI; HIST1H2AK; HIST1H2AL;<br>HIST1H2AM; HIST1H2AG	99% with HIST1H2AJ<br>91% with HIST1H2AH<br>88% with HIST1H2AC<br>88% with HIST2H2AA3<br>88% with HIST2H2AA4<br>88% with HIST1H2AD<br>86% with HIST3H2A	H2A.1, H2A/p, histone H	100% with H2A1H<br>99% with H2A1D<br>99% with H2A1J<br>98% with H2A2A<br>98% with H2A1B<br>98% with H2A1C<br>98% with H2A3<br>98% with H2A.J<br>97% with H2A2C<br>95% with H2AX<br>95% with H2A2B<br>93% with H2A1A<br>67% with H2A.V
	HIST1H2AA		H2A1A, H2A/r	96% with H2A2B<br>95% with H2A1J<br>95% with H2A1H<br>94% with H2A1C<br>93% with H2AX<br>93% with H2A2A<br>93% with H2A.1<br>93% with H2A2C<br>93% with H2A3<br>92% with H2A1D<br>92% with H2A1B<br>91% with H2A.J<br>67% with H2A.V<br>67% with mH2A1<br>66% with mH2A2
	HIST1H2AE; HIST1H2AB	87% with HIST1H2AH<br>86% with HIST1H2AK<br>85% with HIST1H2AG<br>85% with HIST1H2AJ<br>85% with HIST1H2AC<br>85% with HIST1H2AI<br>85% with HIST1H2AM	H2A type 1-B/E, H2A.2, H	99% with H2A1D<br>99% with H2A3<br>98% with H2A.1<br>98% with H2A1C<br>98% with H2A1H<br>98% with H2A1J<br>97% with H2A2A<br>96% with H2A.J<br>95% with H2A2C<br>94% with H2AX<br>92% with H2A1A<br>67% with H2A.V
		90% with HIST1H2AL		99% with H2A3

histone cluster 1	HIST1H2AC	90% with HIST1H2AI<br>88% with HIST1H2AM<br>88% with HIST1H2AK<br>88% with HIST1H2AJ<br>87% with HIST1H2AG<br>87% with HIST1H2AH<br>85% with HIST1H2AD	H2A1C, H2A/l	98% with H2A2A<br>98% with H2A1B<br>98% with H2A.1<br>98% with H2A1H<br>98% with H2A1D<br>98% with H2A1J<br>98% with H2A.J<br>97% with H2A2C<br>96% with H2AX<br>95% with H2A2B<br>94% with H2A1A<br>67% with H2A.V<br>66% with mH2A1
	HIST1H2AD	89% with HIST1H2AL<br>89% with HIST1H2AG<br>88% with HIST1H2AM<br>88% with HIST1H2AK<br>86% with HIST3H2A<br>86% with HIST1H2AI<br>85% with HIST1H2AC<br>85% with HIST2H2AA3<br>85% with HIST2H2AA4	H2A1D, H2A.3, H2A/g	99% with H2A1B<br>99% with H2A.1<br>99% with H2A1H<br>98% with H2A3<br>98% with H2A1J<br>98% with H2A2A<br>98% with H2A1C<br>97% with H2A.J<br>96% with H2A2C<br>94% with H2AX<br>94% with H2A2B<br>92% with H2A1A<br>68% with H2A.V
	HIST1H2AH	91% with HIST1H2AI<br>91% with HIST1H2AG<br>91% with HIST1H2AM<br>91% with HIST1H2AJ	H2A1H, H2A/s	99% with H2A1D<br>99% with H2A1J<br>98% with H2A2A<br>98% with H2A1B<br>98% with H2A1C<br>98% with H2A2C<br>98% with H2A.J
	HIST1H2AJ	99% with HIST1H2AM<br>99% with HIST1H2AI<br>92% with HIST1H2AG<br>91% with HIST1H2AH<br>91% with HIST1H2AK<br>90% with HIST1H2AL<br>88% with HIST1H2AC<br>87% with HIST2H2AA3<br>87% with HIST2H2AA4<br>87% with HIST3H2A	H2A1J, H2A/e	99% with H2A1H<br>99% with H2A.1<br>98% with H2A1D<br>98% with H2A2C<br>98% with H2A2A<br>98% with H2A1B<br>98% with H2A1C<br>97% with H2A3<br>97% with H2A.J<br>95% with H2A2B<br>95% with H2A1A<br>95% with H2AX<br>67% wih H2A.V
		95% with HIST2H2AC<br>88% with IST1H2AI		98% with H2A.1<br>98% with H2A1C

	HIST2H2AA4; HIST2H2AA	87% with HIST1H2AJ<br>86% with HIST2H2AB<br>86% with HIST1H2AG<br>86% with HIST1H2AH<br>86% with HIST1H2AM<br>85% with HIST1H2AD<br>85% with HIST1H2AL	H2A2A, H2A/o	98% with H2A2C<br>98% with H2A1H<br>98% with H2A1D<br>98% with H2A3<br>98% with H2A1J<br>98% with H2A.J<br>97% with H2A1B<br>95% with H2AX<br>95% with H2A2B<br>93% with H2A1A<br>68% with H2A.V<br>67% with mH2A1<br>66% with mH2A2
histone cluster 2	HIST2H2AB	87% with HIST2H2AC<br>87% with HIST2H2AA4<br>86% with HIST2H2AA3	H2A2B	98% with H2A2C<br>97% with H2A.J<br>96% with H2A1A<br>96% with H2AX<br>95% with H2A1C<br>95% with H2A1H<br>95% with H2A1J<br>95% with H2A2A<br>95% with H2A.1<br>95% with H2A3<br>94% with H2A1D<br>94% with H2A1B<br>68% with mH2A1<br>67% with mH2A2<br>67% with H2A.V
	HIST2H2AC	95% with HIST2H2AA4<br>95% with HIST2H2AA3<br>87% with HIST1H2AJ<br>87% with HIST2H2AB<br>87% with HIST2H2AI<br>86% with HIST1H2AG<br>86% with HIST1H2AM<br>85% with HIST1H2AC<br>85% with HIST1H2AH	H2A2C, H2A/q, H2A-GL10	98% with H2A2A<br>98% with H2A1H<br>98% with H2A1J<br>98% with H2A2B<br>97% with H2A.1<br>97% with H2A1C<br>97% with H2A.J<br>96% with H2A1D<br>96% with H2A3<br>95% with H2A1B<br>95% with H2AX<br>93% with H2A1A<br>68% with H2A.V<br>67% with mH2A1<br>66% with mH2A2
		88% with H2AFX<br>87% with HIST1H2AL<br>87% with HIST1H2AI<br>87% with HIST1H2AJ<br>87% with HIST1H2AC		99% with H2A1B<br>99% with H2A1C<br>98% with H2A1D<br>98% with H2A2A<br>98% with H2A.1

histone cluster 3	HIST3H2A	86% with HIST1H2AK<br>86% with HIST1H2AG<br>86% with HIST1H2AD	H2A3	98% with H2A1H<br>97% with H2A1J<br>97% with H2A.J<br>96% with H2A2C<br>95% with H2AX<br>95% with H2A2B<br>93% with H2A1A<br>68% with H2A.V<br>66% with mH2A1

*: homology above 85%

**: homology above 66%

Supplemantary Table 2: Nucleotide and amino acid sequence homology of mouse H2A family members

MOUSE				
	Nucleotide		Protein	
Histones	Name of coding gene(s)	Homology (above 85%)	Name (s)	Homology (above 66%)
H2A histone family, member Y	H2afy		mH2A1, macroH2A1, H2A.y,	68% with H2AX<br>68% with H2A2B<br>67% with mH2A2<br>67% with H2A2C<br>67% with H2A2A<br>66% with H2A3<br>66% with H2A.J
H2A histone family, member Y2	H2afy2		mH2A2, macroH2A2	68% with H2A2B<br>67% with mH2A1<br>66% with H2A2C<br>66% with H2A2A
H2A histone family, member J	H2afj	92% with Hist1h2ai<br>92% with Hist1h2ah<br>92% with Hist1h2ao<br>92% with Hist1h2ad<br>92% with Hist1h2ag<br>91% with Hist1H2an<br>91% with Hist1h2af<br>91% with Hist1h2ac<br>91% with Hist1h2ab<br>91% with Hist1h2ak<br>91% with H2afx<br>89% with Hist2H2ac	H2A.J, H2a/j	98% with H2A3<br>97% with H2A2A<br>97% with H2A1H<br>97% with H2A1F<br>96% with H2A2C<br>96% with H2A2B<br>96% with H2A1K<br>94% with H2AX<br>66% with mH2A1
H2A histone family, member V	H2afv		H2A.V, H2A.F/Z	98% with H2A.Z<br>66% with H2A2C<br>66% with H2A2A<br>66% with H2A3
H2A histone family, member Z	H2afz	no similarity	H2A.Z	98% with H2A.V
H2A histone family, member X	H2AFX	92% with Hist1h2ai<br>92% with Hist1h2ak<br>91% with Hist1H2ag<br>91% with Hist1h2ad<br>91% with Hist1H2ah<br>91% with Hist1h2an<br>91% with Hist1h2ae<br>90% with Hist1h2af<br>90% with Hist2h2aa2<br>90% with H2afj	H2AX, H2A.X, H2a/x	94% with H2A2B<br>94% with H2A2A<br>94% with H2A2C<br>94% with H2A.J<br>94% with H2A3<br>94% with H2A1K<br>93% with H2A1H<br>93% with H2A1F<br>68% with mH2A1<br>66% with mH2A2
H2A histone family, member B1	h2afb1		H2A-Bbd type 1, H2a Barr body deficient	
		99% with Hist1h2ad<br>98% with Hist1h2ao<br>98% with hist1h2an<br>98% with Hist1h2ag		99% with H2A1H<br>99% with H2A1<br>98% with H2A1K<br>98% with H2A3

histone cluster 1	Hist1h2af	98% with Hist1h2ah<br>98% with Hist1h2ae<br>98% with Hist1h2ai<br>97% with Hist1h2ab<br>97% with Hist1h2ac<br>97% with Hist1h2ak<br>93% with Hist2h2ac<br>91% with Hist2h2aa2<br>91% with Hist1h2aa1	H2A1F	
	Hist1h2ab; Hist1h2ac; Hist1h2ad; Hist1h2ae;	98% with Hist1h2ah<br>97% with Hist1h2af<br>97% with Hist1h2ae<br>97% with Hist1h2ak<br>92% with Hist2h2ac<br>91% with Hist2h2aa2	H2A1, h2a type-1	100% with H2A1H<br>99% with H2A1K<br>99% with H2A3<br>99% with H2A1F<br>97% with H2A2A<br>97% with H2A.J<br>95% with H2A2C<br>93% with H2A2B
	Hist1h2ah	99% with Hist1h2ai<br>99% with Hist1h2ac<br>99% with Hist1h2ag<br>99% with Hist1h2ao<br>99% with Hist1h2ad<br>99% with Hist1h2an<br>98% with Hist1h2ae<br>98% with Hist1h2ab<br>98% with Hist1h2af<br>97% with Hist1h2ak<br>92% with Hist2h2ac<br>92% with Hist2h2aa2<br>92% with Hist2h2aa1	H2A1H	100% with H2A1<br>99% with H2A1K<br>99% with H2A3<br>99% with H2A1F<br>97% with H2A2A<br>97% with H2A2C<br>97% with H2A.J<br>94% with H2A2B<br>93% with H2AX
	Hist1h2ak	98% with Hist1h2ao<br>98% with Hist1h2ae<br>97% with Hist1h2ai<br>97% with Hist1h2ad<br>97% with Hist1h2ah<br>97% with Hist1h2an<br>97% with Hist1h2ac<br>97% with Hist1h2ab<br>97% with Hist1h2af<br>94% with Hist1h2ag<br>92% with H2afx<br>91% with Hist2h2aa2<br>91% with Hist1h2aa1	H2A1K	99% with H2A1<br>99% with H2A1H<br>98% with H2A1F<br>98% with H2A3<br>96% with H2A2A<br>96% with H2A.J<br>95% with H2A2C<br>94% with H2AX<br>92% with H2A2B
		97% with Hist2h2ac<br>92% with Hist1h2ao		98% with H2A2C<br>98% with H2A3

histone cluster 2	Hist2h2aa1, hist2h2aa2	92% with Hist1h2ai<br>92% with Hist1h2ag<br>92% with Hist1h2ad<br>92% with Hist1h2ac<br>90% with H2afx<br>92% with Hist1h2ah<br>91% with Hist1h2ae<br>91% with Hist1h2an<br>91% with Hist1h2ak<br>91% with Hist1h2ab<br>91% with Hist1h2af	H2A2A, H2A.2,	97% with H2A1<br>97% with H2A1H<br>97% with H2A.J<br>96% with H2A1K<br>96% with H2A1F<br>95% with H2A2B<br>94% with H2AX<br>67% with mH2A<br>66% with mH2A2<br>66% wihth H2A.V
	hist2h2ab	89% with Hist2h2ac<br>89% with Hist2h2aa2<br>89% with Hist2h2aa1<br>88% with Hist1h2ad<br>87% with Hist1h2ag<br>87% with Hist1h2ai<br>87% with Hist1h2af<br>87% with Hist1h2ae<br>87% with Hist1h2ao<br>87% with Hist1h2ah<br>87% with Hist1h2an<br>87% with Hist1h2ac<br>87% with Hist1h2ab	H2A2B, H2a-613A	98% with H2A2C<br>96% with H2A.J<br>95% with H2A2A<br>94% with H2AX<br>94% with H2A3<br>94% with H2A1H<br>93% with H2A1<br>93% with H2A1F<br>92% with H2A1K<br>68% with mH2A1<br>68% with mH2A2
	hist2h2ac	97% with Hist2h2aa2<br>97% with Hist2h2aa1<br>93% with Hist1h2ag<br>93% with Hist1h2ad<br>93% with Hist1h2ao<br>93% with Hist1h2af<br>93% with Hist1h2ae<br>92% with Hist1h2ac<br>92% with Hist1h2ah<br>92% with Hist1h2an<br>92% with Hist1h2ab<br>92% with Hist1h2ai<br>91% with Hist1h2ak	H2A2C, H2a-613B	98% with H2A2A<br>98% with H2A2B<br>97% with H2A1H<br>97% with H2A1F<br>96% with H2A3<br>96% with H2A.J<br>95% with H2A1<br>95% with H2A1K<br>94% with H2AX<br>67% with mH2A1<br>66% with mH2A2<br>66% with H2A.V
histone cluster 3	hist3h2a	90% with Hist1h2ai<br>90% with Hist1h2ac<br>89% with Hist1h2ah<br>89% with Hist1h2ao<br>89% with Hist1h2ag<br>89% with Hist1h2ad<br>89% with Hist1h2an<br>88% with Hist1h2af<br>88% with Hist1h2ab<br>87% with Hist1h2ak<br>86% with Hist2h2aa2<br>86% with Hist1h2aa1	H2A3	99% with H2A1<br>99% with H2A1H<br>98% with H2A1K<br>98% with H2A1F<br>98% with H2A2A<br>98% with H2A.J<br>96% with H2A2C<br>94% with H2AX<br>94% with H2A2B<br>66% with mH2A1<br>66% with H2A.V

*: homology above 85%

**: homology above 66%
